# A Sex-Specific Analysis of Nutrition Label Use and Health, Douglas County, Nebraska, 2013

**DOI:** 10.5888/pcd12.150167

**Published:** 2015-09-24

**Authors:** Dejun Su, Junmin Zhou, Hannah L. Jackson, Ghada A. Soliman, Terry T-K Huang, Amy L. Yaroch

**Affiliations:** Author Affiliations: Junmin Zhou, Hannah L. Jackson, Ghada A. Soliman, University of Nebraska Medical Center, Omaha, Nebraska; Terry T-K Huang, University of Nebraska Medical Center and City University of New York, New York, New York; Amy L. Yaroch, University of Nebraska Medical Center and Gretchen Swanson Center for Nutrition, Omaha, Nebraska.

## Abstract

**Introduction:**

In 2014 the US Food and Drug Administration proposed a series of changes to its 1992 guidelines on nutrition facts labeling to help consumers make informed food choices. To date, few studies have examined the association between consumers’ use of the nutrition label and health. The objective of this study was to assess the association between nutrition label use and health and to determine whether the association differs by sex.

**Methods:**

Using data from a population-based, random sample survey of 1,503 participants conducted in Nebraska in 2013, we performed χ^2^ tests to examine bivariate associations between selected health variables and nutrition label use, followed by logistic regression analysis to estimate these associations in a multivariate framework.

**Results:**

A U-shaped relationship between self-rated health (SRH) and nutrition label use was observed. Both excellent and poor SRH were associated with a higher likelihood of nutrition label use than the 3 SRH categories in between. Being obese or having 1 of 4 chronic conditions (hypertension, diabetes, heart disease, high cholesterol) were both associated with higher odds of nutrition label use (odds ratio [OR] = 2.63, *P* < .001; OR = 1.71, *P* < .05, respectively) among men. These associations, however, were not significant among women.

**Conclusion:**

A close association existed between health and nutritional label use. This association was more pronounced among men than among women. Nutrition education may benefit from factoring in the association between health and use of nutrition labels and the differences in these associations by sex.

## Introduction

The 1990 Nutrition Labeling and Education Act mandated that standardized nutrition information appear on all packaged foods (1). In 2014, the US Food and Drug Administration (FDA) proposed a series of changes to the 1990 guidelines to help consumers make informed food choices to support healthy eating and lifestyles. The changes aimed to improve consumers’ understanding of nutrition and food science, updated serving size requirements, and changed the design of the label (2). Although it is too soon to tell if the changes are accomplishing what FDA intended, findings from numerous studies have consistently shown that reading a nutrition label is associated with healthy food choices (3–7).

Consumers may use nutrition labels for different reasons. Some may use the nutrition information to aid in the consumption of more healthful foods and overall chronic disease prevention, whereas others may have chronic diseases and have been advised by their doctors to follow certain nutrition or dietary guidelines (8–10). Few studies to date have assessed the association between nutrition label use and health. Identifying this association and the factors contributing to label use could lead to a better understanding of the impact of nutrition labels on food choices among people with various health needs.

In this study, we analyzed data from a population-based survey to assess whether nutrition label use was associated with health among adults in Douglas County, Nebraska (Omaha area), and how it was associated. Because previous literature suggested that women were more likely to read nutrition labels than men (11,12), we sought to explore the association between health and nutrition label use for each sex. Men have a higher risk of cardiovascular diseases and associated death than women (13–19), and diet is a factor in preventing and treating cardiovascular diseases. We examined 4 hypotheses: 1) that a U-shaped relationship exists between self-rated health and nutrition label use such that excellent and poor health are both associated with a high likelihood of nutrition label use relative to health categories in between (very good, good, fair); 2) that being obese is related to a higher probability of using nutrition labels relative to being nonobese; 3) that having a chronic disease is positively related to nutrition label use; and 4) that substantial sex differences exist in the association between health and use of nutrition labels.

## Methods

### Data

Data for this study were from the Douglas County Community Health Survey, a population-based telephone survey conducted in the summer of 2013. The survey’s target population was residents aged 18 years or older in Douglas County, the largest and most demographically diverse county in Nebraska with a 2013 estimated population of 524,697, of which approximately 11% were Hispanic, and 11% African American. Another factor in selection of Douglas County was its proximity to the research and survey teams. The sampling frame of the survey was based on telephone numbers generated through the Genesys Sampling Systems, 2013 version (Marketing Systems Group), providing a comprehensive coverage of both landline and cellular telephones eligible for the survey with an oversampling of minority and rural residents. The use of standard random-digit dialing and computer-assisted telephone interviewing technique made it possible for the survey to generate a probability sample in which analytical results could be generalized to the study area. Altogether 1,503 participants (729 men and 774 women) completed the survey in either English (95.3%) or Spanish (4.7%). The overall response rate, combining both landline and cellular telephone interviews, was 39.8%. The data were weighted using a 3-step process of calculating design weights, adjusting for nonresponse, and then raking to match the sample to population totals. This study was approved by the institutional review board of University of Nebraska Medical Center. Informed consent was obtained from each participant before administering the survey.

### Measures

Nutrition label use was assessed with the following question: “Do you pay attention to information about sodium, fat, calories, or the use of preservatives when you purchase food?” Participants were given 5 possible responses: 1) always, 2) usually, 3) sometimes, 4) rarely, 5) never. The responses were further dichotomized into 2 categories: 1) usually not (sometimes, rarely, and never), and 2) usually do (all other responses).

In our analysis we examined the use of nutrition labels in relation to 5 variables: health status, demographics, socioeconomic status (SES), health behavior, and health care access. Health status variables were self-rated health (excellent, very good, good, fair, or poor), obesity (body mass index [BMI] ≥30), self-reported weight change in the past year (no change, gained weight, and lost weight), and measures on chronic conditions (whether the participant had ever been told by a doctor he or she had 1 of 4 diseases: hypertension, heart disease, diabetes, or high cholesterol). We also created a dummy variable with 1 indicating having at least 1 or more of the 4 chronic conditions, and 0 indicating having none.

Variables on demographics and SES were age, sex, marital status (married vs unmarried), race/ethnicity (non-Hispanic white, black, Hispanic, or other) country of birth (United States vs other), employment (employed vs unemployed), and education. All variables were based on self-report.

Health behavior and lifestyle variables were measured by smoking status (smoked at least 100 cigarettes in your life or never smoked), alcohol consumption (consumed alcohol in the past 30 days or not), leisure-time physical activity, number of meals eaten at fast food restaurants per week (continuous variable), and dietary preference (prefer meat or vegetables/fruits or no preference). Health care access was defined as having health insurance coverage and having at least 1 personal doctor.

### Statistical analysis

We conducted bivariate cross-tabulations to assess the association between nutrition label use and selected health indicators. We used χ^2^ statistics and related *P* values to determine whether associations were significant. We then conducted 3 logistic regression analyses in which we examined the relationship between nutrition label use and health indicators after controlling for selected variables on demographics, SES, health behaviors, and other selected variables of the whole sample, and of males and females. All analyses were conducted with the weighted sample in SPSS, version 21 (IBM Corp).

## Results

### Differences by sex

Several differences between men and women were significant (Table 1). Men were less likely to use nutrition labels than were women (40.7% vs 54.3%, *P* < .001). Men were also less likely than women to report having gained weight over the past year (15.9% vs 29.1%, *P* < .001)). The proportion of male respondents who were employed was higher than the proportion of women (72.7% vs 61.6%, *P* < .001). Men were less likely than women to report having at least 1 personal doctor (69.5% vs 81.2%, *P* < .001).

**Table 1:** Variables Used in Analysis of Nutrition Label Use and Health in a Sample (N = 1,503) of Men and Women, Douglas County, Nebraska, 2013

Variables	Total Sample, N^a^	Men, n^a^	Women, n^a^	Difference Between Sexes, χ^2^
**Dependent variable**				
Pay attention to information about sodium, fat, calories, or the use of preservatives when purchasing food				
Usually do not	780 (52.3)	430 (59.3)	350 (45.7)	27.69^b^
Usually do	711 (47.7)	295 (40.7)	416 (54.3)	
**Independent variables**				
**Health-related variables**				
**Self-rated health**				
Excellent	328 (21.8)	153 (21.0)	175 (22.6)	16.68^c^
Very good	556 (37.0)	299 (41.0)	257 (33.2)	
Good	396 (26.4)	171 (23.4)	226 (29.2)	
Fair	169 (11.2)	88 (12.1)	81 (10.4)	
Poor	53 (3.5)	18 (2.4)	35 (4.6)	
**Obesity**				
Not obese	996 (70.3)	506 (70.4)	490 (70.2)	0.01
Obese	421 (29.7)	213 (29.6	208 (29.8)	
**Weight change since last year**				
No change	701 (46.9)	381 (52.2)	320 (41.8)	38.46^b^
Gained weight	339 (22.6)	116 (15.9)	223 (29.1)	
Lost weight	455 (30.5)	233 (31.9)	223 (29.1)	
**Has chronic conditions**				
No	900 (60.5)	432 (59.9)	467 (61.0)	0.20
Has at least one of 4 conditions (hypertension, heart disease, diabetes, high cholesterol)	588 (39.5)	289 (40.1)	298 (39.0)	
**Hypertension**				
No	1,100 (73.2)	542 (74.3)	558 (72.0)	0.90
Yes	403 (26.8)	187 (25.7)	215 (27.9)	
**Heart disease**				
No	1,386 (92.6)	665 (91.4)	721 (93.6)	2.55
Yes	111 (7.4)	62 (8.6)	49 (6.4)	
**Diabetes**				
No	1,352 (89.9)	645 (88.4)	707 (91.3)	3.4^d^
Yes	151 (10.1)	84 (11.6)	67 (8.7)	
**High cholesterol**				
No	1,159 (77.6)	555 (76.8)	604 (78.5)	0.61
Yes	334 (22.4)	168 (23.2)	166 (21.5)	
**Demographic characteristics**				
Age, mean (% [SD])^e^	1,483 (44.6 [18.0])	721 (44.0 [18.0])	763 (45.2 [18.0])	—
**Sex**				
Male	729 (48.5)	—	—	—
Female	774 (51.5)	—	—	
**Marital status**				
Unmarried	759 (50.8)	345 (47.4)	415 (54.1)	6.74^c^
Married	734 (49.2)	383 (52.6)	352 (45.9)	
**Race/ethnicity**				
Non-Hispanic white	1,084 (73.1)	552 (75.9)	532 (70.3)	6.05
Black	178 (12.0)	77 (10.6)	101 (13.3)	
Hispanic	163 (11.0)	72 (9.9)	91 (12.0)	
Other	59 (4.0)	26 (3.5)	33 (4.4)	
**Country of birth**				
United States	1,343 (89.7)	654 (89.9)	689 (89.4)	0.09
Other	155 (10.3)	73 (10.1)	81 (10.6)	
**Socioeconomic status**				
**Employment**				
Unemployed	493 (33.0)	199 (27.3)	294 (38.4)	20.77^b^
Employed	1,001 (67.0)	529 (72.7)	471 (61.6)	
**Education**				
High school or less	502 (33.6)	240 (33.0)	262 (34.1)	3.65
Some college	460 (30.8)	240 (33.0)	220 (28.6)	
College or above	534 (35.7)	247 (34.0)	287 (37.3)	
**Health behavior**				
**Smoking status**				
Nonsmoker	899 (60.1)	445 (61.1)	454 (59.1)	0.63
Smoker	597 (39.9)	283 (38.9)	314 (40.9)	
**Alcohol consumption**				
No drinking	567 (38.1)	249 (34.5)	318 (41.4)	7.45^c^
Drinking	922 (61.9)	472 (65.5)	450 (58.6)	
**Leisure-time physical activity**				
No	160 (10.7)	95 (13.0)	65 (8.5)	8.23^c^
Yes	1,338 (89.3)	634 (87.0)	704 (91.5)	
**Number of meals eaten at fast food restaurants per week, mean (% [SD])^f^**	1,469 (2.3 [3.2])	709 (2.8 [3.7])	761 (1.8 [2.6])	—
**Dietary preference**				
Prefer meat	422 (28.2)	283 (38.8)	139 (18.1)	114.73^b^
Prefer vegetables/fruits	359 (23.9)	103 (14.1)	256 (33.2)	
No preference	718 (47.9)	343 (47.1)	375 (48.7)	
**Health care access**				
Has personal doctor				
No	362 (24.5)	219 (30.5)	143 (18.8)	26.97^b^
At least one doctor	1,114 (75.5)	459 (69.5)	615 (81.2)	
**Health insurance coverage**				
Insured	1,291 (86.6)	623 (85.9)	669 (87.3)	0.64
Uninsured	199 (13.4)	102 (14.1)	97 (12.7)	

Abbreviation: SD, standard deviation; —, not applicable.

a Values in parentheses are means unless otherwise indicated.

b *P* < .001.

c *P* < .01.

d *P* < .10.

e For difference between the sexes, *t* = 1.23.

f For difference between the sexes, *t* = 6.05, *P* < .001.

Diet patterns also differed between men and women; men reported a higher frequency of fast-food consumption than did women (2.8 vs 1.8 times per week, *P* < .001). In addition, men were more likely to report a preference for meat than women (38.8% vs 18.1%). By contrast, women were more likely to prefer fruits and vegetables than men (33.2% vs 14.1%, *P* < .001).

### Bivariate associations

Overall, bivariate associations between health and nutrition label use were more pronounced among men than women (Table 2). For example, although having hypertension was significantly associated with a higher use of nutrition labels among men (χ^2^ =18.53, *P* < .001), this association was not significant among female respondents. Similar differences between men and women were also observed for reported diabetes and high cholesterol.

**Table 2:** Bivariate Associations Between Nutrition Label Use and Selected Health Indicators Among Sample (N = 1,503) of Men and Women, Douglas County, Nebraska, 2013

Do you pay attention to information about sodium, fat, calories, or the use of preservatives when you purchase food?												
Response	Total Sample, n (%)		Total	*χ* ^2^	Male, n (%)		Total	*χ* ^2^	Female, n (%)		Total	*χ* ^2^
	Usually Not	Usually Do			Usually Not	Usually Do			Usually Not	Usually Do		
**Hypertension**												
No	597 (54.8)	492 (45.2)	1,089 (100.0)	9.91^a^	344 (63.9)	194 (36.1)	538 (100.0	18.53^b^	253 (45.9)	298 (54.1)	551 (100.0)	0.02
Yes	183 (45.6)	218 (54.4)	401 (100.0)		86 (46.0)	101 (54.0)	187 (100.0)		97 (45.3)	117 (54.7)	214 (100.0)	
Total	780 (52.3)	710 (47.7)	1,490 (100.0)		430 (59.3)	295 (40.7)	725 (100.0)		350 (45.8)	415 (54.2)	765 (100.0)	
**Heart disease**												
No	744 (54.1)	630 (45.9)	1,374 (100.0)	22.77^b^	408 (61.7)	253 (38.3)	661 (100.0)	20.38^b^	336 (47.1)	377 (52.9)	713 (100.0)	6.34^c^
Yes	34 (30.6)	77 (69.4)	111 (100.0)		20 (32.3)	42 (67.7)	62 (100.0)		14 (28.6)	35 (71.4)	49 (100.0)	
Total	778 (52.4)	707 (47.6)	1,485 (100.0)		428 (59.2)	295 (40.8)	723 (100.0)		350 (45.9)	412 (54.1)	762 (100.0)	
**Diabetes**												
No	708 (52.7)	636 (47.3)	1,344 (100.0)	0.73	392 (60.9)	252 (39.1)	644 (100.0)	5.74^c^	316 (45.1)	384 (54.9)	700 (100.0)	1.00
Yes	72 (49.0)	75 (51.0)	147 (100.0)		37 (46.8)	42 (53.2)	79 (100.0)		34 (51.5)	32 (48.5)	66 (100.0)	
Total	780 (52.3)	711 (47.7)	1,491 (100.0)		429 (59.3)	294 (40.7)	723 (100.0)		350 (45.7)	416 (54.3)	766 (100.0)	
**High cholesterol**												
No	616 (53.7)	531 (46.3)	1,147 (100.0)	5.52^c^	342 (62.1)	209 (37.9)	551 (100.0)	9.36^a^	274 (46.0)	322 (54.0)	596 (100.0)	0.16
Yes	155 (46.4)	179 (53.6)	334 (100.0)		82 (48.8)	86 (51.2)	168 (100.0)		73 (44.2)	92 (55.8)	165 (100.0)	
Total	771 (52.1)	710 (47.9)	1,481 (100.0)		424 (59.0)	295 (41.0)	719 (100.0)		347 (45.6)	414 (54.4)	761 (100.0)	
**Obesity**												
No	524 (52.8)	468 (47.2)	992 (100.0)	1.47	317 (62.6)	189 (37.4)	506 (100.0)	10.90^a^	206 (42.5)	279 (57.5)	485 (100.0)	2.72^d^
Yes	205 (49.3)	211 (50.7)	416 (100.0)		103 (49.3)	106 (50.7)	209 (100.0)		102 (49.3)	105 (50.7)	207 (100.0)	
Total	729 (51.8)	679 (48.2)	1,408 (100.0)		420 (58.7)	295 (41.3)	715 (100.0)		308 (44.5)	384 (55.5)	692 (100.0)	
**Self-rated health**												
Excellent	132 (40.2)	196 (59.8)	328 (100.0)	63.23^b^	74 (48.4)	79 (51.6)	153 (100.0)	23.27^b^	58 (33.1)	117 (66.9)	175 (100.0)	63.20^b^
Very good	292 (52.9)	260 (47.1)	552 (100.0)		195 (65.0)	105 (35.0)	300 (100.0)		98 (38.6)	156 (61.4)	254 (100.0)	
Good	226 (57.4)	168 (42.6)	394 (100.0)		103 (60.2)	68 (39.8)	171 (100.0)		123 (55.2)	100 (44.8)	223 (100.0)	
Fair	115 (71.4)	46 (28.6)	161 (100.0)		55 (65.5)	29 (34.5)	84 (100.0)		61 (78.2)	17 (21.8)	78 (100.0)	
Poor	13 (24.5)	40 (75.5)	53 (100.0)		4 (22.2)	14 (77.8)	18 (100.0)		9 (25.7)	26 (74.3)	35 (100.0)	
Total	778 (52.3)	710 (47.7)	1,488 (100.0)		431 (59.4)	295 (40.6)	726 (100.0)		349 (45.6)	416 (54.4)	765 (100.0)	
**Weight change**												
No change	425 (60.7)	275 (39.3)	700 (100.0)	47.16^b^	251 (65.9)	130 (34.1)	381 (100.0)	20.39^b^	174 (54.5)	145 (45.5)	319 (100.0)	26.71^b^
Gained weight	175 (52.1)	161 (47.9)	336 (100.0)		70 (60.9)	45 (39.1)	115 (100.0)		105 (47.5)	116 (52.5)	221 (100.0)	
Lost weight	180 (40.0)	270 (60.0)	450 (100.0)		108 (47.4)	120 (52.6)	228 (100.0)		71 (32.1)	150 (67.9)	221 (100.0)	
Total	780 (52.5)	706 (47.5)	1,486 (100.0)		429 (59.3)	295 (40.7)	724 (100.0)		350 (46.0)	411 (54.0)	761 (100.0)	

a *P* < .01

b *P* < .001.

c *P* < .05

d *P* < .10

Among men with heart disease, 67.7% reported nutrition label use compared with 38.3% of men without heart disease (χ^2^ = 20.38, *P* < .001). The corresponding percentages among females were 71.4% and 52.9%, respectively (*P* < .05). Obese males reported more nutrition label use (50.7%) compared with nonobese males (37.4%) (*P* < .01). The opposite was shown for females: 50.7% of obese females and 57.5% of nonobese females (*P* < .10) reported nutrition label use.

Results indicated a U-shaped relationship between self-reported health status and nutrition label use among both men and women. For both men and women, self-reported poor and excellent health were both associated with higher likelihood of nutrition label use compared with other self-rated health categories (*P* < .001).

Respondents who reported changes in body weight during the previous year were more likely to have higher nutrition label use than those who reported no change since the previous year (*P* < .001). Notably, losing weight was associated with the highest nutrition label use. Among women, 67.9% of those who lost weight reported nutrition label use as compared with 45.5% among their counterparts who did not experience weight change. Similar findings were observed among men. 

### Multivariate analyses

We used a multivariate model to assess the relationship between health and nutrition label use, controlling for sociodemographic factors, health behaviors, and health care access (Table 3). The bivariate U-shaped relationship of the association between SRH and nutrition label use was confirmed in multivariate analysis. However, relative to what was found among women, this association was more significant among the entire sample and among men. For example, men who reported excellent health had higher odds of using nutrition labels than those who reported very good health (54% lower [*P* < .01]) and good health (60% lower [*P* < .01]). The corresponding effect among women, however, was not significant.

**Table 3:** Multivariate Logistic Regression on Nutrition Label Use Among Sample (N = 1,503) of Men and Women, Douglas County, Nebraska, 2013

Variable	Total Sample (n = 1,253)	Male (n = 539)	Female (n = 714)
	Odds Ratio (95% CI)			
**Health-related variables**			
**Self-rated health**			
Excellent	1 [Reference]		
Very good	0.58^a^ (0.41–0.81)	0.46^a^ (0.28–0.74)	0.72 (0.43–1.22)
Good	0.56^a^ (0.38–0.82)	0.40^a^ (0.22–0.72)	0.69 (0.39–1.21)
Fair	0.22^b^ (0.13–0.38)	0.56 (0.27–1.17)	0.06^b^ (0.03–0.16)
Poor	2.16^c^ (0.90–5.18)	3.47^c^ (0.88–13.70)	1.59 (0.46–5.52)
**Obesity**			
No	1 [Reference]		
Obese	1.81^b^ (1.33–2.46)	2.63^b^ (1.69–4.09)	1.28 (0.79–2.05)
**Weight change since last year**			
No change	1 [Reference]		
Gained weight	1.69^a^ (1.22–2.34)	1.41 (0.84–2.39)	2.78^b^ (1.72–4.48)
Lost weight	2.72^b^ (2.02–3.67)	2.55^b^ (1.68–3.87)	3.87^b^ (2.37–6.31)
**Has chronic conditions**			
No	1 [Reference]		
Has at least 1 of 4 conditions (hypertension, heart disease, diabetes, high cholesterol)	1.21 (0.88–1.66)	1.71^d^ (1.10–2.66)	0.94 (0.56–1.58)
**Demographics**			
**Age**	1.01^a^ (1.00–1.02)	1.01 (0.99–1.02)	1.02^a^ (1.01–1.04)
**Sex**			
Male	1 [Reference]		
Female	1.14 (0.87–1.49)	—	—
**Marital status**			
Unmarried	1 [Reference]		
Married	1.42^d^ (1.07–1.88)	1.25 (0.82–1.92)	1.59^d^ (1.04–2.44)
**Race/ethnicity**			
Non-Hispanic white	1 [Reference]		
Black	1.20 (0.78–1.84)	1.39 (0.72–2.71)	1.40 (0.74–2.65)
Hispanic	1.36 (0.79–2.32)	0.82 (0.35–1.91)	1.61 (0.74–3.54)
Other	1.95^c^ (0.97–3.90)	1.76 (0.62–5.06)	3.48^d^ (1.21–10.05)
**Country of birth**			
United States	1 [Reference]		
Other	0.51^d^ (0.29–0.90)	0.49 (0.21–1.15)	0.57 (0.25–1.32)
**Socioeconomic status**			
**Employment**			
Unemployed	1 [Reference]		
Employed	1.39^d^ (1.02–1.90)	1.53 (0.92–2.54)	1.55^c^ (0.99–2.41)
**Education**			
High school or less	1 [Reference]		
Some college	1.31 (0.94–1.83)	1.01 (0.61–1.69)	1.52 (0.91–2.55)
College degree or above	1.88^b^ (1.33–2.67)	1.47 (0.85–2.55)	2.84^b^ (1.68–4.81)
**Health behavior**			
**Smoking status**			
Nonsmoker	1 [Reference]		
Smoker	0.92 (0.70–1.20)	0.88 (0.60–1.30)	1.06 (0.70–1.60)
**Alcohol consumption**			
No drinking	1 [Reference]		
Drinking	0.89 (0.67–1.20)	0.83 (0.54–1.28)	1.05 (0.67–1.64)
**Leisure-time physical activity**			
No	1 [Reference]		
Yes	1.64^d^ (1.03–2.63)	3.22^a^ (1.64–6.32)	0.60 (0.28–1.29)
**Dietary preference**			
Number of meals eaten at fast food restaurants per week	0.92^b^ (0.88–0.96)	0.92^a^(0.87–0.98)	0.89^a^(0.82–0.96)
Prefer meat	1 [Reference]		
Prefer vegetables/fruits	4.44^b^(3.03–6.50)	4.88^b^ (2.57–9.28)	6.08^b^ (3.46–10.69)
No preference	1.25 (0.92–1.69)	1.27 (0.84–1.93)	1.85^d^ (1.11–3.09)
**Health care access**			
Has a personal doctor			
No	1 [Reference]		
At least one doctor	1.18 (0.83–1.66)	1.85^d^ (1.14–3.02)	0.74 (0.42–1.30)
**Health insurance coverage**			
Insured	1 [Reference]		
Uninsured	0.95 (0.61–1.48)	1.48 (0.79–2.78)	0.85 (0.41–1.78)

Abbreviations: CI, confidence interval; —, not applicable.

a *P* < .01.

b *P* < .001.

c *P* < .10.

d *P* < .05.

Similar findings were also observed for obese respondents and for respondents with at least 1 of 4 chronic conditions (hypertension, heart disease, diabetes, or high cholesterol). In the total sample, obesity was associated with a higher nutrition label use (odds ratio [OR] = 1.81; *P* < .001). This association was even more evident among men (OR = 2.63; *P* < .001). By contrast, the corresponding association among women was not significant. We also found that although having at least 1 of the 4 chronic conditions considered was associated with higher nutrition label use among men (OR = 1.71; *P* < .05), the association was not significant among women. Relative to those who did not experience weight change since last year, respondents who lost weight during the past year had higher odds of nutrition label use (OR = 2.72; *P* < .001), followed by those who gained weight (OR = 1.69, *P* < .01). A similar association was observed among women. However, the corresponding association was less pronounced among men where gaining weight was not significantly associated with nutrition label use.

Findings also indicated associations between certain demographic characteristics and nutrition label use. In general, the probability of label reading increased with age, especially for women (OR = 1.02; 95% confidence interval [CI], 1.01–1.04; *P* < .01). (We treated the variable age as continuous, so for every 1 year increase in age, we expected to see about a 2% increase in the odds of label reading.) Being married was associated with a higher probability of label reading than being unmarried in the total sample as well as among women (*P* < .05 in both cases). Country of birth was linked to label reading in the total sample. Immigrants were less likely than US-born respondents to read nutrition labels (OR = 0.51; 95% CI, 0.29–0.90, *P* < .05).

SES was associated with nutrition label use in the total sample and among women. Being employed was associated with a higher probability of label use in the whole sample (OR = 1.39; 95% CI, 1.02–1.90, *P* < .05) but was not significant for men. Similarly, having a college education or more was associated with the highest odds of label use in the total sample and among women (*P* < .001 in both cases), but the effect was not significant among men.

We found a substantial difference between sexes in the association between leisure-time physical activity and label use. Among men, the odds for those who had leisure-time physical activity to read labels were much higher compared with those reporting no leisure-time physical exercise (OR = 3.22; 95% CI, 1.64–6.32, *P* < .01). This association, however, was not observed in women.

Dietary pattern and preference was significantly associated with nutrition label use among both men and women. The number of fast food meals consumed each week was negatively associated with the nutrition label use (*P* < .01 for both men and women). Compared with respondents who preferred meat in their diet, respondents who expressed a preference for fruits and vegetables were more likely to use nutrition labels, both men (OR = 4.88; *P* < .001) and women (OR = 6.08; *P* < .001). One notable difference was that among women only, expressing no preference between meat and fruits and vegetables turned out to be associated with higher nutrition label use than expressing preference for meat (OR = 1.85; *P* < .05).

Although we did not observe a significant association between health insurance coverage and nutrition label use, we did observe an association between having at least 1 personal doctor or not with label reading among men. Men who had at least 1 personal doctor were more likely than men who had no personal doctor to read labels is significantly higher (OR = 1.85; *P* < .05). The association, however, was not observed among women in the sample.

## Discussion

This study assessed correlates of nutrition label use and how these correlates differ for men and women in a random sample of residents in Douglas County, Nebraska. Overall, the results indicated a close association between nutrition label use, health outcomes, and health behavior. We observed a U-shaped relationship between SRH and nutrition label use; excellent and poor SRH were both associated with a higher odds of nutrition label use than the 3 SRH categories between them. Nutrition label use was also associated with leisure-time physical activity and preference for vegetables and fruits. Despite this general pattern, the association between health and nutrition label use was more pronounced among men than among women. Being obese or having one of the 4 chronic conditions considered (hypertension, heart disease, diabetes, high cholesterol) were both associated with higher odds of nutrition label use among men. These associations, however, were not significant among females.

These findings highlight important differences between the sexes in use of nutrition labels and plausible factors contributing to the differences. Consistent with related findings from previous studies (11,12), women were more likely than men to report nutrition label use when purchasing food. One explanation is that traditional gender roles might encourage women to be meticulous about food selection for the whole family (20). However, the 2 groups might be using nutrition labels for different reasons. The closer linkages between health and nutrition label use among men may be suggestive of unique medical needs of men. This can be partially corroborated by our observation that men who had at least 1 personal doctor were more likely to read nutrition labels than those who did not. By contrast, women were more likely than men to be dissatisfied with body image and to have stronger motivation to change body weight by modifying their diet (21,22). Our findings indicated that for both men and women, weight change since the previous year, relative to no change, was associated with higher odds of nutrition label use; however, this association seems more pronounced among women than among men. This partially explains why obesity was associated with nutrition label use only among men, but not among women, once the effect of body weight change had been taken into account. Similar findings were also reported among adolescents where results indicated that reading nutrition labels was associated with higher fat intake among boys, but not among girls (23).

An implication of these findings is that nutrition education efforts may need to be tailored to specific populations (eg, men vs women, younger vs older) and to modes of delivery. For instance, the strong association between chronic conditions and nutrition label use among men tends to suggest that the clinical setting might be a potential place to disseminate targeted nutrition education for men. Similarly, other potential settings to disseminate this type of information to men can include gymnasiums and parks, given the close association between physical exercises and nutrition label use among males.

This study has several limitations. First, the use of cross-sectional data made it difficult for us to infer causality. Second, the survey data we used contained no qualitative data on nutrition label use such as the purpose of reading the label, perceived usefulness of the label, and how label use impacts dietary behavior. Moreover, the survey question on nutrition label use concerned only sodium, calories, fat, or use of preservatives and did not cover all nutrition elements. Third, the use of self-reported data can potentially incur recall bias especially for variables based on respondents' long-term memory, such as changes in body weight since the previous year. Some of the question items in the survey were not tested for reliability and validity. Finally, this study was based on a regional sample in Nebraska, which limits generalizability of our findings to other regions.

Despite these limitations, this population-based study is unique in that it assessed the less-examined associations between nutrition label use and health status, especially exploring differences among men versus women. These findings add to the extant body of literature on differences between men and women in health behaviors and reinforce justifications for sex-specific nutrition education (24). Future studies could confirm these findings in a large, nationally representative sample and explore how tailored interventions could be developed to increase nutrition label use and improve dietary patterns across various populations.
